# Topological Electric Field-Defined Quantum Dots in Bilayer Graphene: An Atomistic Approach

**DOI:** 10.3390/ma19132790

**Published:** 2026-07-01

**Authors:** Włodzimierz Jaskólski

**Affiliations:** Institute of Physics, Faculty of Physics, Astronomy and Informatics, Nicolaus Copernicus University in Toruń, Grudziądzka 5, 87-100 Toruń, Poland; wj@fizyka.umk.pl

**Keywords:** multilayer graphene, quantum dots, topological states

## Abstract

We study topological bound states in quantum dots defined by an electric field in bilayer graphene. An external field is perpendicular to the bilayer, and changes sign in a finite region that defines the quantum dot. The electric field opens a gap in the bilayer graphene, and the reversed field creates a domain wall with one-dimensional chiral gapless bands localized therein. The finite size of dots leads to quantization of these bands and the appearance of discrete bound states localized at the dot boundary. We consider rectangular dots oriented along the armchair and zigzag directions. We go beyond a simple continuum one-valley model and use an atomistic tight-binding approach. This allows us to identify new effects related to the atomic structure of graphene and the strengths of the electric field, as well as the valley mixing, valley position, and valley asymmetry.

## 1. Introduction and Model Description

There have been a series of theoretical works over the last few years devoted to study of the energy structure of electric field-defined topological quantum dots in bilayer graphene (BLG QD) [1,2,3]. On the one hand, these works are in response to the significant progress that has been made recently in the experimental realization and manipulation of various BLG QDs [4,5,6,7,8,9,10,11]. On the other hand, they are part of the growing interest in graphene-based zero-dimensional structures due to their potential applications, e.g., in quantum information processing [12,13,14,15,16,17,18,19,20,21,22,23,24,25,26].

A perpendicular electric field applied to Bernal-stacked bilayer graphene opens a tunable energy gap. A spatially varying field can confine electrons in finite regions of the BLG. The most intriguing configuration is when the field changes only the sign in different regions. In such a case, a domain wall is formed between the regions, at which one-dimensional (1D) chiral gapless modes are localized [27]. When the region where the field changes its polarization is finite, the 1D modes discretize to a series of bound states localized at the edge of the region. These are called topologically confined states. The cited works [1,2,3], although using only simple one-valley continuum approximations, are the first to calculate these states for various electrically defined quantum dots in BLG.

**Figure 1:**
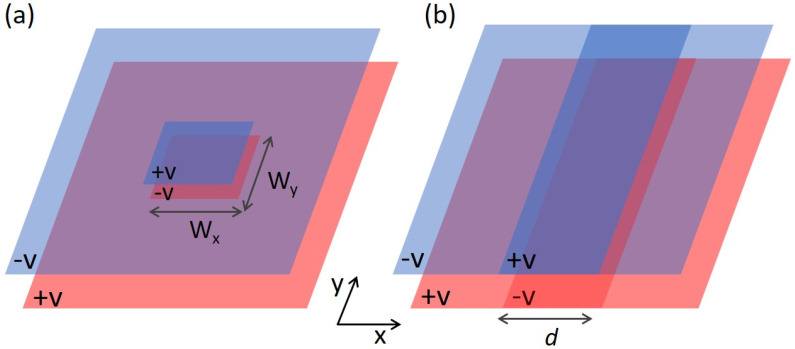
Schematic representations of electrically defined rectangle BLG QD (**a**) and a system of two parallel EFWs separated by *d* (**b**). The dot is defined as a region in a gated BLG, where the voltages ±*V* applied to the layers (red and blue) are reversed.

However, continuum models do not take into account the atomic and periodic structure of the BLG, do not recognize different directions in graphene, are mostly limited to weak fields, and cannot account for possible valley interaction. This paper aims to go a step forward, to fill this gap and calculate the energy structure of electrically defined rectangular BLG QDs, as schematically visualized in Figure 1a, in an atomistic tight-binding approach (TB). To investigate the influence of the BLG atomic structure on edge-confined states in electrically defined QDs, we perform calculations for rectangular dots oriented in the armchair and zigzag directions. This is, however, challenging, because electrically defined BLG QDs of ∼50 nanometers in size would require that calculations be performed on systems comprising almost half a million atoms.

To address this challenge, we simplify the model and begin by examining two parallel electric-field domain wells (EFWs) created by reversing the electric field twice, as shown schematically in Figure 1b. First, infinite-length domain walls with one-dimensional topological bands localized at the walls are considered. Then, walls of finite length are taken into account, leading to band discretization and the formation of topologically confined bound states. The parallel walls act as opposite sides of the rectangular QD. The 1D topological bands localized at perpendicular sides of the QD discretize independently, which simplifies the calculations. The TB calculations reveal several new effects that are not accessible in simpler models. We find that the bound states localized at the zigzag edges are affected by the cone position and asymmetry. This makes them behave differently from the states localized at the armchair edges as the dot size increases. We also show that on the zigzag edges bound states with energies independent of the QD size can additionally appear.

## 2. Computational Methods

The Hamiltonian in the π-electron tight-binding approximation that we use in calculations is

$$H=t_{i/e}\sum_{<i,j>}c_{i}^{†}c_{j}+H.c.$$

where $c_{i}^{†}$ and ($c_{i}$) are the creation and (annihilation) operators for electrons at site *i*, and symbol <*i*,*j*> restricts summation to the nearest neighbors. We use the standard intralayer and inter-layer hopping parameters *t_i_* = 2.7 eV and *t_e_* = 0.27 eV [28,29]. The boundary conditions (BCs) applied in the calculations for different structures are discussed in the sections where the results are presented.

The perpendicular electric field is considered by adding gate voltages ±*V* to the upper and lower BLG layers. The calculations are performed for two different gates, *V* = ±0.1 V and *V* = ±0.5 V. In the first case, the voltage applied between the layers is smaller than the interlayer interaction energy *t_e_*, while in the second case it is larger. This choice is dictated by previously observed gate control of the layer localization of gapless states at domain walls formed by the stacking order change in multilayer graphene [28,30]. It may influence band crossing in the case of two parallel domain walls.

In a realistic experiment, the change in the potential at the domain wall is rather smooth. A smooth potential can also model screening of the applied electric field. However, as discussed in Ref. [2], a smooth potential results in more densely spaced energy branches compared to the case of an abrupt potential change. Therefore, because the aim of this work is to show that the atomistic approach yields some new results compared to continuum one-valley approximations, the domain walls are, for simplicity, formed by a simple sign reversal of the voltage applied. Also, we do not investigate the effects of perturbations like disorder or imperfections; these are beyond the scope of the present study. However, the robustness of topological states at stacking domain walls in multilayer graphene to strong perturbations has been shown in Refs. [31,32].

## 3. Results and Discussion

### 3.1. Two Parallel EFWs

First, we investigate the system of two parallel EFWs, as schematically shown in Figure 1b. The domain walls can extend along the armchair or zigzag directions and are separated by a distance *d* measured in the number of unit cells in a given direction. The unit cells contain eight carbon atoms (four in each layer). The lengths of unit cells in the zigzag and armchair directions are *a_z_* = √3*a_C_*_–*C*_ and *a_a_* = 3*a_C_*_–*C*_, respectively, where *a_C_*_–*C*_ is the distance between two carbon atoms in a layer. To calculate the energy bands of such systems, we must impose boundary conditions away from the domain walls. The BCs can be open (the edges in the *x*-direction of the entire system shown in Figure 1b are not connected) or closed (edges connected). We choose the size of the system in the direction perpendicular to the EFWs, large enough to ensure that the results are practically independent of the size and the BC. In cases of EFWs in the zigzag direction, we use closed BC to get rid of zigzag edge states.

**Figure 2:**
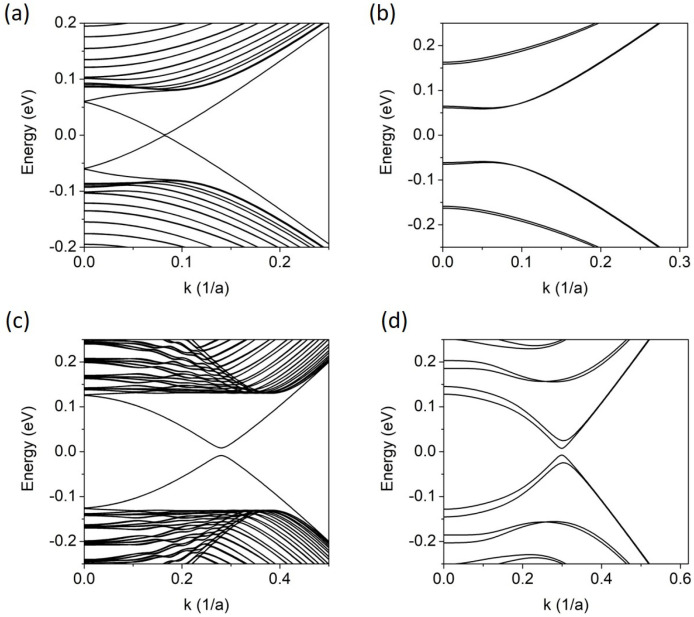
Energy bands close to Γ (*k* = 0) for two parallel EFWs along the armchair direction. In (**a**,**c**), the walls are separated by 140 unit cells; in (**b**,**d**), they are separated by 20 unit cells. Voltages applied to the layers are *V* = ±0.1 V in (**a**,**b**), and *V* = ±0.5 V in (**c**,**d**).

Each domain wall in a system of two parallel EFWs introduces into each valley (Dirac cone) a pair of topological one-dimensional gapless states of a given slope (momentum). If the EFW lies along the armchair direction, the valleys *K* and *K′* overlap at the Γ point, so even for a single EFW there are two pairs of gapless bands, each with an opposite slope. Thus, when two EFWs are very well separated, all these bands are two-fold degenerate, and the crossing point is four-fold degenerate. This is seen in Figure 2a for *d* = 140 *a_z_* and the low voltage *V* = 0.1 V. The higher voltage *V* = 0.5 V acts as a strong perturbation and removes the degeneracy of the pair of bands at the same domain wall, i.e., corresponding to *K* and *K′* (see Figure 2c at *k* ≈ 0.3), but the bands maintain two-fold degeneracy due to the large separation distance between the domain walls. When the separation distance is small, *d* = 20 *a_z_* (Figure 2b,d), the topological bands slightly split and anti-cross due to the interaction of bands localized at different domain walls.

**Figure 3:**
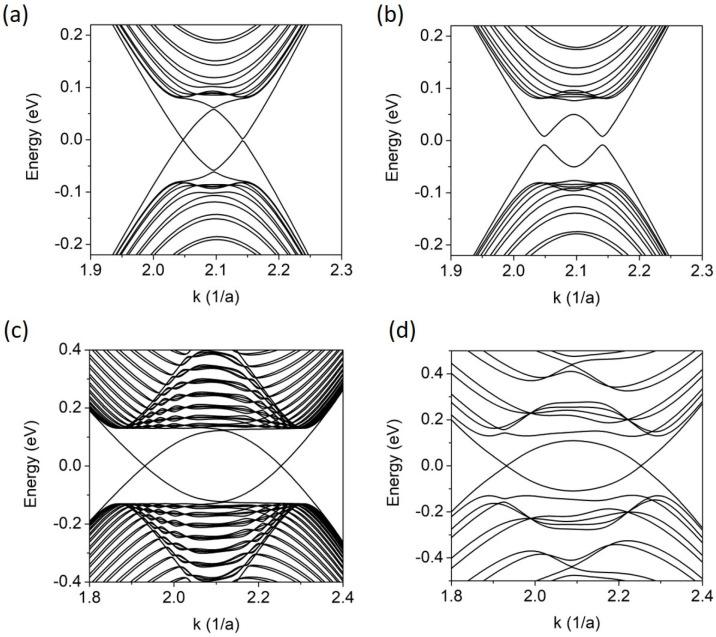
Energy bands close to *k* = 2*π*/(3*a_z_*) for two parallel EFWs along the zigzag direction. In (**a**,**c**), the walls are separated by 80 unit cells; in (**b**,**d**), they are separated by 20 unit cells. Voltage applied to the layers are *V* = ±0.1 V in (**a**,**b**), and *V* = ±0.5 V in (**c**,**d**).

The energy bands of two EFWs along the zigzag direction, separated by *d* = 80 and *d* = 40 unit cells for the two gates *V* = 0.1 V and *V* = 0.5 V, are shown in Figure 3. In this case, all the 1D gapless bands are nondegenerate, because they correspond to a single *K*; the pairs of bands with opposite slopes originate from different domain walls. For *V* = 0.1 V and a smaller separation distance between the EFWs (*d* = 20 *a_a_*, Figure 3b), the topological bands slightly anti-cross. However, for *V* = 0.5 V, the bands cross even for the small *d* = 20 *a_a_*. This is because at high voltages the crossing bands localize mainly in different layers and non-connected nodes of the layers [28].

### 3.2. Two Parallel EFWs of Finite Width

In this section, we investigate a double EFW of finite length *W*, which we call the width to be consistent with the description of finite-size QD (see Figure 1a). To see how the topological bands are discretized for a domain wall of finite width, we select the discrete values of the wave vector *k* in the calculations performed in the previous section, i.e.,

(1) $$k_{n}=\frac{n\pi }{W},\;n=1,\;2,\;\ldots ;N\;$$

where *W* = *Na_a/z_* and *a_a/z_* equals *a_a_* or *a_z_* for EFW in the armchair or zigzag direction, respectively. This is equivalent to closed BC applied at *W* [33]. Those *E_n_* that lie within the energy gap correspond to discrete energy levels arising from topological gapless modes. The corresponding wave functions are localized at the EFWs.

**Figure 4:**
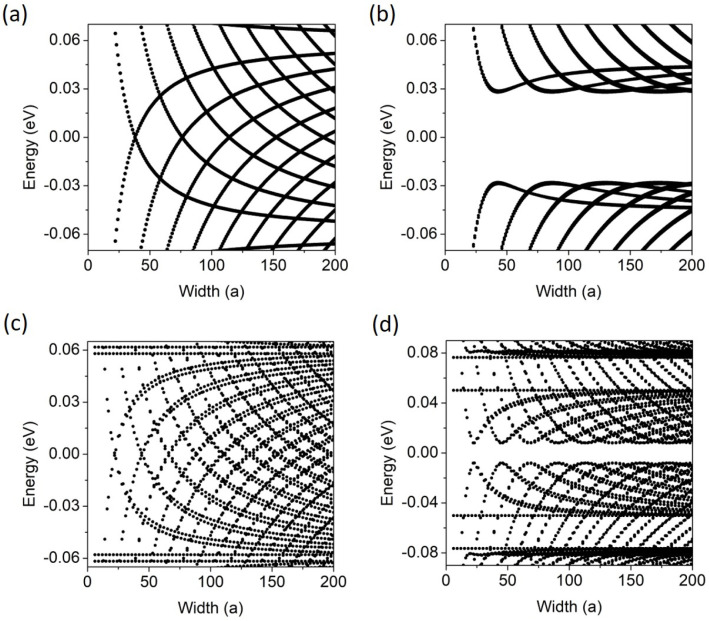
Energy levels of two parallel EFWs of finite width *W*. The voltages applied to the layers are *V* = ±0.1 V. Upper panels: EFWs along the armchair direction, separated by 160 unit cells (**a**) and by 40 unit cells (**b**). Lower panels: EFWs along the zigzag direction, separated by 80 and 40 unit cells in (**c**) and (**d**), respectively.

The discrete energy levels of two EFWs along the armchair direction, as a function of the domain wall width *W*, for *V* = 0.1 V and two separations between the walls, *d* = 160 *a_z_* and *d* = 40 *a_z_*, are shown in Figure 4a and Figure 4b, respectively. For each *d*, the energy levels arrange in kinds of branches for increasing *W* [2,3]. The branches cross, and have mirror symmetry with respect to *E* = 0, the same as the 1D gapless bands in Figure 2. For a small separation between the domain walls, *d =* 40 *a_z_*, the absolute energy gap appears. For very large widths *W*, the branches converge to the energies corresponding to *k* = 0 in the case of infinitely long domain walls (i.e., *E* ≈ ±0.6 eV in Figure 2a for *d* = 140 *a_z_*). It is also worth noting that all the discrete energy levels are doubly degenerate, as are their corresponding 1D topological bands.

The discrete energy levels of two finite EFWs along the zigzag direction, as a function of the domain wall width *W*, for *V* = 0.1 V and two separations between the walls, *d* = 80 *a_a_* and *d* = 40 *a_a_*, are shown in Figure 4c and Figure 4d, respectively. Although the general pattern of the energy branches is similar to that of the two EFWs along the armchair direction, there are important differences that need to be analyzed and explained. These main differences are: (i) the appearance of flat branches, and (ii) the duplication of branches that looks like splitting. As for the flat branches, they result from the position of the Dirac cone, which, in the zigzag direction, is at *k* = 2π/(3*a_z_*). Thus, the flat branches appear whenever the width *W* = *Na_z_* is a multiple of 3 in Equation (1), i.e., *N* = 3*M*, where *M* is an integer. In such a case, the cut of topological bands always yields the same energy for all *n* = 2/3*N*. This does not happen when EFW is in the armchair direction, because even for a large *W*, we never reach the cone center at *k* = 0.

As for branch duplication, this is not a true splitting, because the topological bands for EFW in the zigzag direction are not degenerate. This effect results from the cone position and its asymmetry. In the zigzag direction, the cone is at *k* = 2π/(3*a_z_*). Therefore, the consecutive values of *k_n_*, which are spaced by π/(*Na_z_*) (Equation (1)), are not symmetrically distributed around the cone. As a consequence, the energy levels corresponding to the pairs of *k_n_* and *k_n’_* (for the same or the consecutive values of *N*) on the left and right sides of the cone may have close, but different values. This effect is additionally enhanced by the cone asymmetry, which is an intrinsic property of the valley when the graphene *k*-space is projected along the line corresponding to the zigzag direction. The slope of *E*(*k*_0_ + *δk*) is different from the slope *E*(*k*_0_ − *δk*). It is like moving on the graphene 2D energy surface from *K* to *M* on one side of *k*_0_ and from *K* to Γ on the other. This cone asymmetry is clearly seen in Figure 3c,d (the cone asymmetry is more pronounced for higher gates, because of the larger overlap of the valence and conduction band continua). The asymmetry of the band continua translates into an asymmetry of the topological bands, and enhances the effect observed as branch duplication.

It is worth emphasizing that none of these effects would be visible in continuum single-valley approximations, because they essentially reflect only the armchair direction with a symmetric cone.

**Figure 5:**
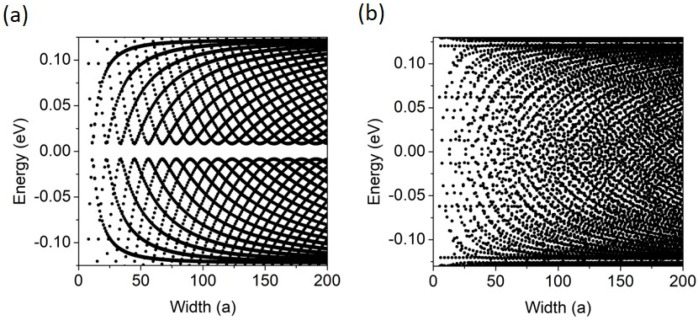
Energy levels of two parallel EFWs of finite width *W*. The voltages applied to the layers are *V* = ±0.5 V. (**a**) EFWs in armchair direction separated by 80 unit cells; (**b**) EFWs in zigzag direction separated by 40 unit cells.

In Figure 5, branches of discrete energy levels for *V* = 0.5 V are presented. Figure 5a shows energy levels of two EFWs of finite width *W* in the armchair direction, separated by *d* = 80 *a_z_*. The absolute energy gap arises from partial splitting of the crossed 1D bands, as described in the previous section and shown in Figure 2c,d. Figure 5b shows energy levels of two EFWs of finite width *W* in the zigzag direction, separated by *d* = 40 *a_a_*. Branch duplication is clearly seen, and flat branches at *E* ≈ ±0.14 V are also visible (the second pair of this branch doublet is already embedded in the continuum, see Figure 3d). Other flat branches, closer to *E* = 0 and less dense, are also seen. These appear when *W* = *Na_z_* is a multiple of another integer, *N* = *mM*, and *k_n_* = *nπ/(mMa_z_)* crosses the 1D topological modes in the energy gap. For example, *m* = 5 gives *k_n_* ≈ 1.9 in the units of 1/*a_z_* (see Figure 3c,d) and the same energy levels, close to *E* ≈ ±0.6 eV for all *n* = 3/5.

### 3.3. Rectangular QD

The TB calculation for electrically defined QDs would require considering a system much larger than the QD itself, i.e., a rectangle embedded in BLG with reversed gates, as shown in Figure 1a. For a quantum dot of the order of a hundred units in each direction, the entire system would contain hundreds of thousands of atoms. We avoid large-scale calculations by building on the findings of the previous sections. The momenta (*k*-vectors) of 1D bands localized at perpendicular edges of QD are also perpendicular; more importantly, they have very different values. The 1D bands at the armchair edge have *k* ≈ 0, while those at the zigzag edge have *k* ≈ 2π/(3*a*). Thus, the discretization of 1D topological gap modes localized at the armchair and zigzag-oriented sides of the rectangle is done independently. This is like the wavefunction factorization used in continuous models [1].

Therefore, by selecting discrete values of *k* defined in Equation (1) for *W* = *W_a_* in the case of two EFWs in the armchair direction separated by *d* = *W_z_*, we get discrete energy levels originating from 1D bands localized at the armchair sides of the rectangular QD *W_a_* × *W_z_*. Similarly, selecting discrete values of *k* for *W* = *W_z_* in the case of two EFWs in the zigzag direction separated by *d* = *W_a_*, we get discrete energy levels originating from 1D bands localized at the zigzag sides of the QD. This is exactly what we have done in the previous section. Figure 4 and Figure 5 show the energy levels of topological bound states on one side of the quantum dot (as a function of the width of that side), while keeping the width *d* of the other side constant.

Now, we consider a rectangular QD whose sides change simultaneously with change in the dot size. Their widths, measured in units of *a_a_* and *a_z_*, are *W_a_* and *W_z_*. For simplicity, we consider *W_a_* = *W_z_*, but because the units *a_a_* and *a_z_* are different, the dot is rectangular in nm.

**Figure 6:**
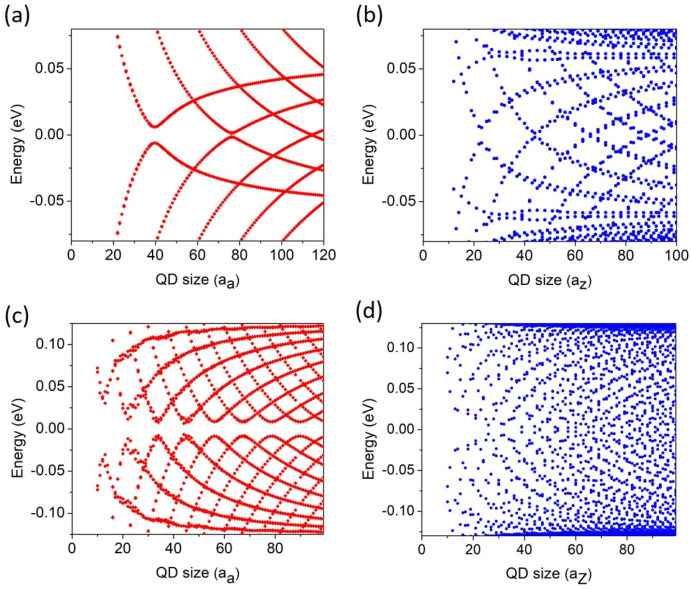
Energy levels of rectangular QDs with edges oriented along the armchair and zigzag directions. Left and right panels show energy levels originating from the armchair and zigzag edges, respectively. Voltages applied to the layers are *V* = ±0.1 in (**a**,**b**), and *V* = ±0.5 V in (**c**,**d**).

In Figure 6 we present the discrete energy levels of a rectangular QD of size *W_a_* × *W_z_* for two different voltages, *V* = 0.1 V in panels (a) and (b), and *V* = 0.5 V in Figure 6c,d. The energy levels corresponding to states localized at the armchair edges are shown in Figure 6a,c; energy levels corresponding to states localized at the zigzag edges are presented in Figure 6b,d (the energy levels originating from different QD edges are presented separately to show their differences). These spectra are similar to those presented in Figure 4 and Figure 5 for two EFWs of finite width. In particular, an absolute energy gap in branches originating from the armchair edge may be seen, as well as doubled branches of bound states localized at zigzag edges. Furthermore, some traces of flat branches are visible in Figure 6b; however, these differ slightly from those in Figure 4 and Figure 5 because the separation distance *d* between the EFWs now changes simultaneously with the width *W*.

## 4. Summary and Conclusions

In the present work, we study electric field-defined rectangular quantum dots in bilayer graphene with the dot sides oriented along the armchair and zigzag directions. Calculations are performed using the tight-binding approach. This allows us to reveal new features of the topological spectra of edge-confined states in electrically defined quantum dots in BLG, compared to simple one-valley continuum approximations.

We begin our analysis by considering two parallel domain walls defined by a change of the field sign. Electric field domain walls introduce and localize a pair of gapless 1D chiral modes, one pair in each valley *K* and *K’*, with opposite valley momenta. For domain walls along the armchair and zigzag directions, the valleys appear at Γ and ±2π/(3*a*), respectively; they also differ in the cone symmetry and degeneracy of the 1D modes. This has a fundamental impact on the spectra of topologically confined states, which arise from the discretization of chiral modes for finite-width domain walls, i.e., the edges of quantum dots.

The energies of topologically confined states arrange themselves into branches as the size of the quantum dot increases. We find that the branches behave differently for bound states localized at the zigzag and armchair edges. The branches of states originating from the zigzag edge are doubled, being a direct consequence of the cone position and asymmetry at 2π/(3*a*). Moreover, when the width *W* of the zigzag edge increases, some energy levels repeat frequently, forming a kind of flat branch. This happens, for example, when *W* is a multiple of 3. It is worth noticing that the edge of an QD of any shape will contain zigzag components; therefore, its spectrum of topologically confined states should also exhibit these features. Finally, we show that the gap opening between the energy branches at the armchair direction can be controlled by the gate voltage *V*.

## Data Availability

The original contributions presented in this study are included in the article. Further inquiries can be directed to the corresponding author.
